# Supplementary material for: Modular biosynthesis of plant hemicellulose and its impact on yeast cells
Source: Biotechnol Biofuels. 2021 Jun 19;14:140. doi: 10.1186/s13068-021-01985-z (PMC8214268; doi:10.1186/s13068-021-01985-z)
Supplement: Supplementary file 1 — Additional file 1: Figure S1. Sanger sequencing alignments of assembled CSL constructs. Figure S2. Genotyping of domain-swapped AtCSLA2/AkCSLA3 yeast colonies. Figure S3. Screening of Pichia colonies expressing recombinant proteins. Table S1. Complete glycosidic linkage table for alkaline-insoluble polymers. Table S2. Enrichment and composition of (gluco)mannan for the top CSLA strains. Figure S4. Oligosaccharide profiling of commercial (gluco)mannan polysaccharides. Figure S5. Yeast cell density and area after CSLA expression. Figure S6. Imaging and quantification of yeast cells stained with Congo Red. Figure S7. Expression of AkCSLA3 in Nicotiana beanthamiana leaves. Figure S8. Replacement of a CSLA catalytic domain with that of a CSLC. Figure S9. Combinatorial effect of AkCSLA3/AtCSLA2 domain swaps. Figure S10. Alignment of active mannan and/or glucomannan synthases. Figure S11. Alignment of CesAs and CSL representatives. Table S3. Primer sequences used in this study. [file 13068_2021_1985_MOESM1_ESM.pdf]

Modular biosynthesis of plant hemicellulose and its impact on yeast cells
DOI: 10.1186/s13068-021-01985-z

Additional file 1

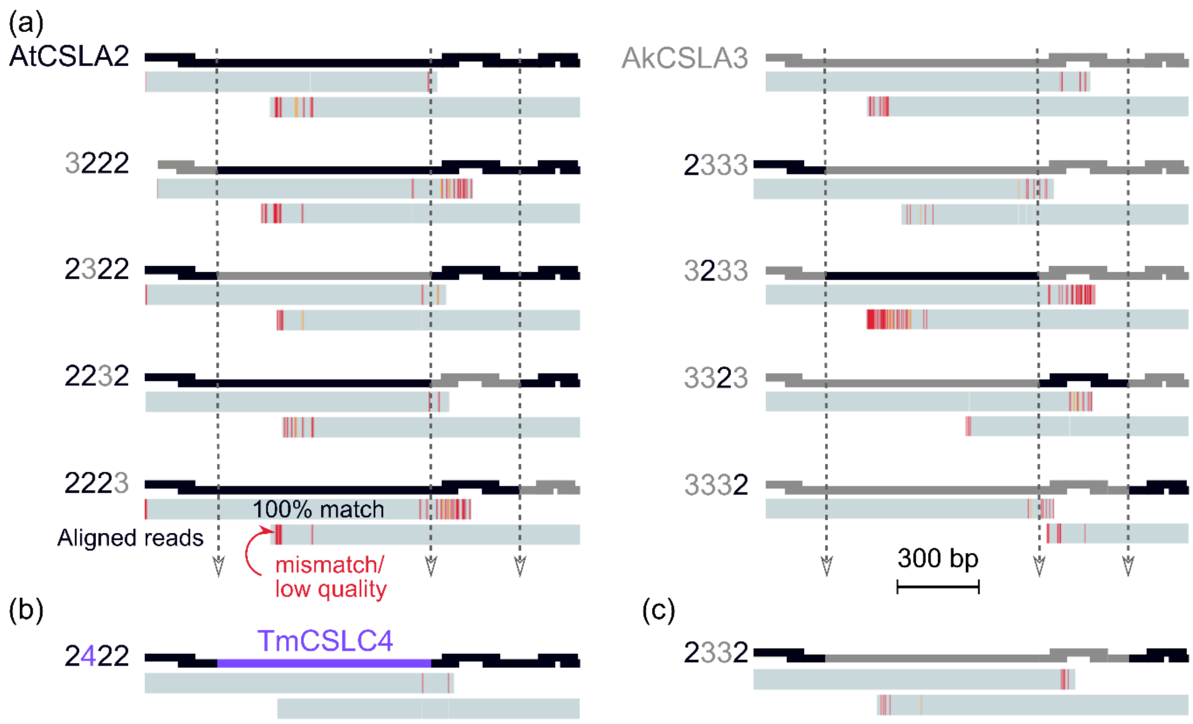

Figure S1. Sanger sequencing alignments of assembled *CSL* constructs.
(a) AtCSLA2/AkCSLA3 single-domain swaps, (b) 2422, and (c) 2332 coding sequences were assembled in the BB1_23 plasmid and verified via overlapping Sanger sequencing reads. Sequences were aligned using Benchling to confirm the desired fusion of the protein domains. Mismatches, displayed in red, are low quality base calls that appear near the end of each read.

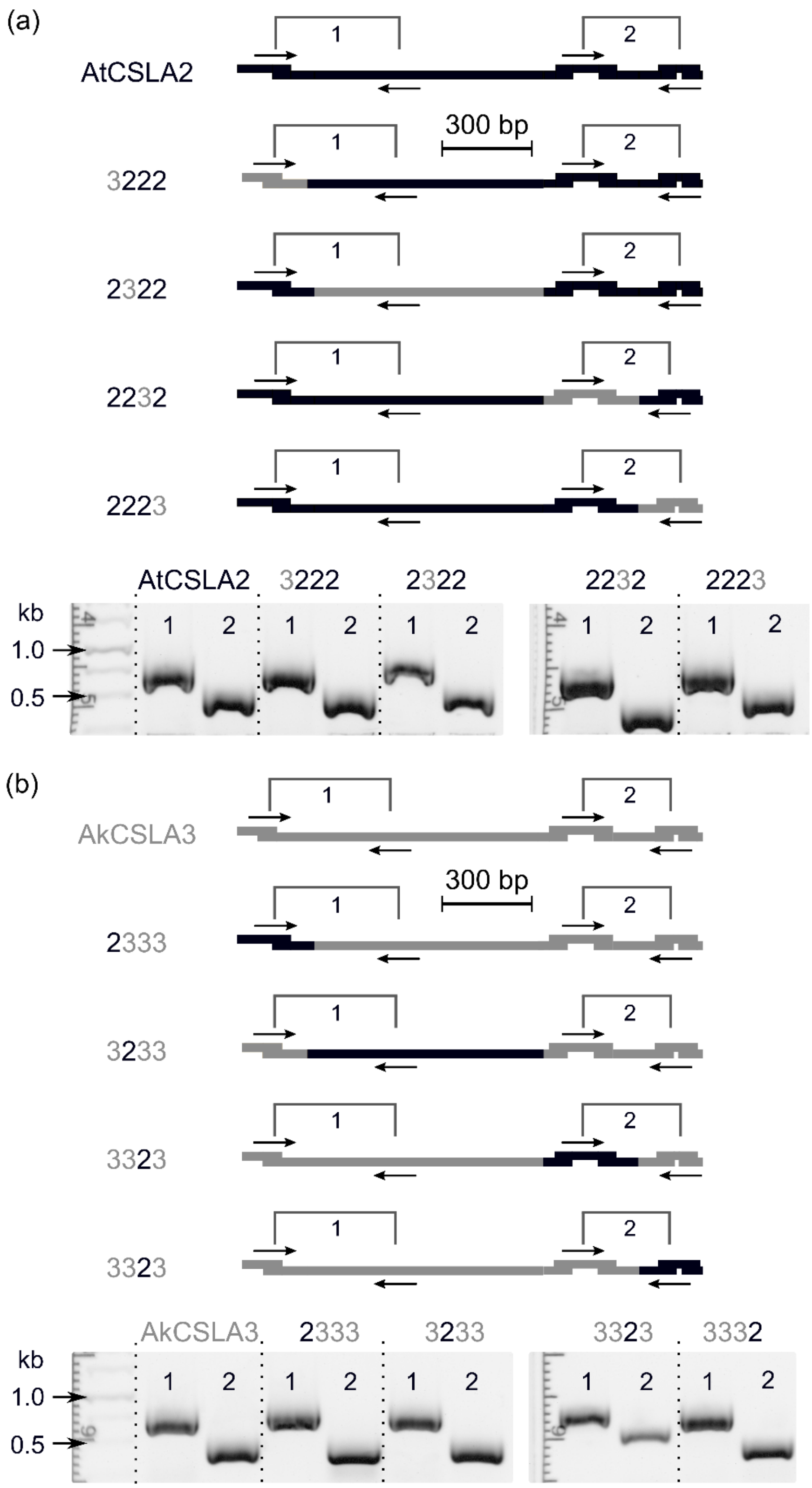

Figure S2. Genotyping of domain-swapped AtCSLA2/AkCSLA3 yeast colonies.
(a) AtCSLA2 and (b) AkCSLA3 parental and domain-swapped transgenes were genotyped following stable integration in the *Pichia pastoris* genome. Colony PCR using two pairs of primers (labelled as 1 and 2) were used to unambiguously verify each construct. The gel shows results for a representative colony for each construct, which were used for further experiments. The first lanes of the gels in (a) and (b) show the distribution of relevant markers from a DNA ladder.

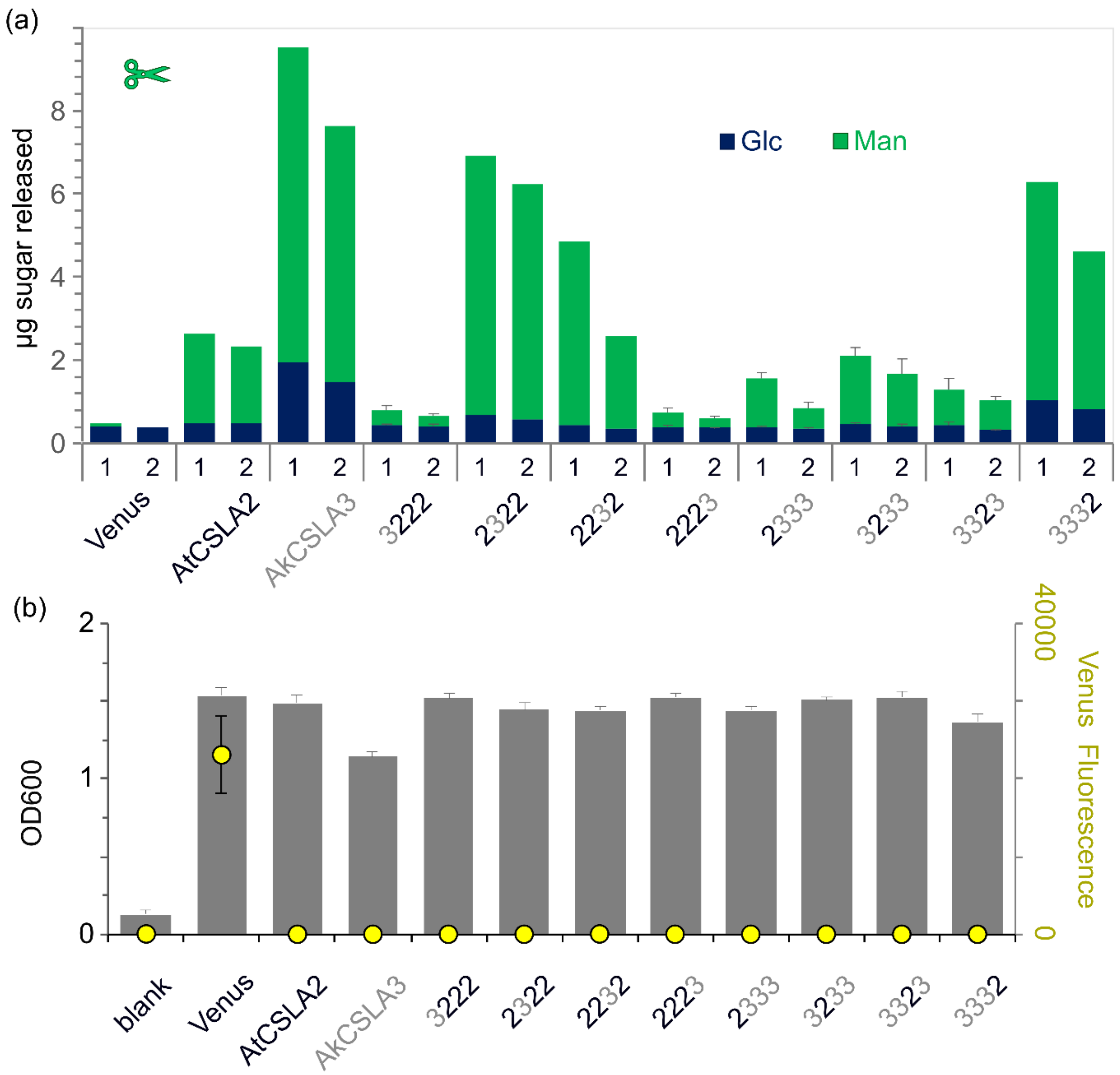

Figure S3. Screening of *Pichia* colonies expressing recombinant proteins.
(a) Screening of (gluco)mannan production following CSLA protein expression in YPM+G for 48 h in *Pichia pastoris*, from the top two independent colonies (labelled 1 and 2) for each construct. Carbohydrates were released from equal aliquots of alkaline-insoluble (AKI) polymers by β-1,4-mannanase digestion. Stacked bars show monosaccharide composition, with the mean + SD of two biological replicates for the least active CSLA strains. (b) The optical density and yellow protein fluorescence of the top colonies (a) following re-growth. Data show mean + SD of four biological replicates for each construct, plus blank medium.

	Venus	AtCSLA2	AkCSLA3	3222	2322	2232	2223	2333	3233	3323	3332
**t-Man**	3.0 ± 0.5	2.7 ± 0.1	1.7 ± 0.1	3.0 ± 0.2	2.2 ± 0.3	1.9 ± 0.1	3.0 ± 0.5	3.1 ± 0.4	2.4 ± 0.1	3.1 ± 0.6	2.2 ± 0.2
**t-Glc**	5.2 ± 0.4	4.2 ± 0.4	4.4 ± 0.1	5.3 ± 0.3	3.6 ± 0.2	3.6 ± 0.3	5.7 ± 0.3	5.0 ± 0.3	4.8 ± 0.3	5.1 ± 0.3	4.2 ± 0.1
**3-Glc**	38.9 ± 1.7	20.5 ± 2.2	27.7 ± 0.6	33.1 ± 1.0	21.2 ± 3.0	17.3 ± 2.4	34.8 ± 2.8	32.2 ± 0.7	30.5 ± 0.8	31.5 ± 0.4	22.9 ± 2.7
**2-Man**	5.2 ± 0.8	3.5 ± 0.1	2.3 ± 0.1	4.5 ± 0.3	2.5 ± 0.6	2.5 ± 0.2	5.0 ± 0.6	4.6 ± 0.4	3.8 ± 0.3	5.0 ± 0.9	2.7 ± 0.3
**4-Man**	0.1 ± 0.0	33.3 ± 8.3	26.7 ± 0.4	4.7 ± 0.2	38.2 ± 1.6	40.2 ± 5.0	4.1 ± 0.8	13.0 ± 1.2	12.0 ± 3.4	6.2 ± 1.9	34.1 ± 2.7
**6-Glc**	26.2 ± 0.9	20.9 ± 3.4	19.3 ± 0.7	29.0 ± 0.8	17.8 ± 2.4	16.1 ± 1.3	26.5 ± 1.3	21.8 ± 1.6	28.4 ± 2.5	29.7 ± 2.4	17.3 ± 3.0
**4-Glc**	3.3 ± 0.4	1.9 ± 0.2	5.2 ± 0.5	2.3 ± 0.1	2.7 ± 0.3	5.4 ± 0.3	2.2 ± 0.1	2.4 ± 0.3	1.7 ± 0.1	1.6 ± 0.1	4.5 ± 0.9
**2,3-Glc**	8.7 ± 1.8	4.5 ± 1.0	5.1 ± 0.5	8.0 ± 0.4	3.9 ± 0.5	4.0 ± 0.5	8.7 ± 1.1	8.5 ± 1.1	6.6 ± 1.1	7.2 ± 1.9	4.2 ± 0.2
**4,6-Man**	0.0 ± 0.0	0.6 ± 0.1	0.3 ± 0.0	0.1 ± 0.0	0.7 ± 0.1	0.9 ± 0.2	0.1 ± 0.0	0.3 ± 0.1	0.2 ± 0.0	0.1 ± 0.0	0.5 ± 0.2
**3,6-Glc**	7.6 ± 0.6	6.6 ± 1.3	6.2 ± 0.1	8.3 ± 0.5	6.1 ± 0.4	6.8 ± 0.7	8.2 ± 0.7	7.4 ± 0.5	8.2 ± 0.8	8.7 ± 0.1	6.2 ± 0.3
**2,6-Man**	1.8 ± 0.3	1.3 ± 0.1	0.9 ± 0.0	1.7 ± 0.1	1.0 ± 0.2	1.3 ± 0.1	1.9 ± 0.3	1.7 ± 0.2	1.4 ± 0.1	1.8 ± 0.3	1.2 ± 0.3

Table S1. Complete glycosidic linkage table for alkaline-insoluble polymers. The percentage of the total glycosidic linkage area for the samples shown in Fig. 3. Data shows the mean ± SD of three biological replicates.

µg sugar AKI	Venus	AtCSLA2	AkCSLA3	2322	2232	3332
**Glc**	955 ± 16	976 ± 31	1,012 ± 36	978 ± 10	939 ± 9	927 ± 23
**Man**	121 ± 4	447 ± 8	673 ± 26	783 ± 19	1,081 ± 46	625 ± 30
µg sugar EM	**Venus**	**AtCSLA2**	**AkCSLA3**	**2322**	**2232**	**3332**
**Glc**	635 ± 2	429 ± 22	747 ± 1	779 ± 27	804 ± 9	921 ± 12
**Man**	85 ± 1	720 ± 10	1,233 ± 14	1,688 ± 58	2,492 ± 71	1,391 ± 34
% Linkage EM	**Venus**	**AtCSLA2**	**AkCSLA3**	**2322**	**2232**	**3332**
**t-Glc / t-Man**	8.1 ± 0.9	5.0 ± 0.1	5.3 ± 0.1	5.8 ± 0.6	5.1 ± 0.9	6.8 ± 0.3
**3-Glc**	46.6 ± 1.5	31.8 ± 0.3	15.9 ± 1.4	26.8 ± 1.3	14.6 ± 1.1	22.1 ± 3.4
**4-Man**	0.0 ± 0.0	36.4 ± 0.5	40.1 ± 2.8	29.9 ± 0.4	51.2 ± 3.1	29.1 ± 5.8
**6-Glc**	30.6 ± 0.8	19.3 ± 0.3	23.2 ± 1.4	26.9 ± 0.6	20.3 ± 0.9	27.4 ± 1.7
**4-Glc**	5.1 ± 0.9	3.0 ± 0.5	9.2 ± 0.5	3.6 ± 0.6	3.6 ± 0.7	7.4 ± 0.7
**3,6-Glc**	9.7 ± 0.7	4.6 ± 0.1	6.3 ± 0.6	7.0 ± 1.6	5.3 ± 0.1	7.2 ± 1.2

Table S2. Enrichment and composition of (gluco)mannan for the top CSLA strains. (Gluco)mannan polymers were sequentially enriched from engineered *Pichia* cells by chemical and enzymatic steps. All data is shown as mean ± SD. The absolute sugar content represents the total AKI content of 2 mL cultures (three biological replicates). Enriched heteromannan (EM) content was measured in duplicate after pooling three AKI samples and removing yeast polymers digested by Zymolyase. EM glycosidic linkages were determined for two technical replicates (only one for AtCSLA2) and were measured in duplicate. The glycosidic linkage percentages shown here are the full dataset for the plot in Fig. 4b. Terminal (t) hexose linkages could not be fully separated in this experimental batch.

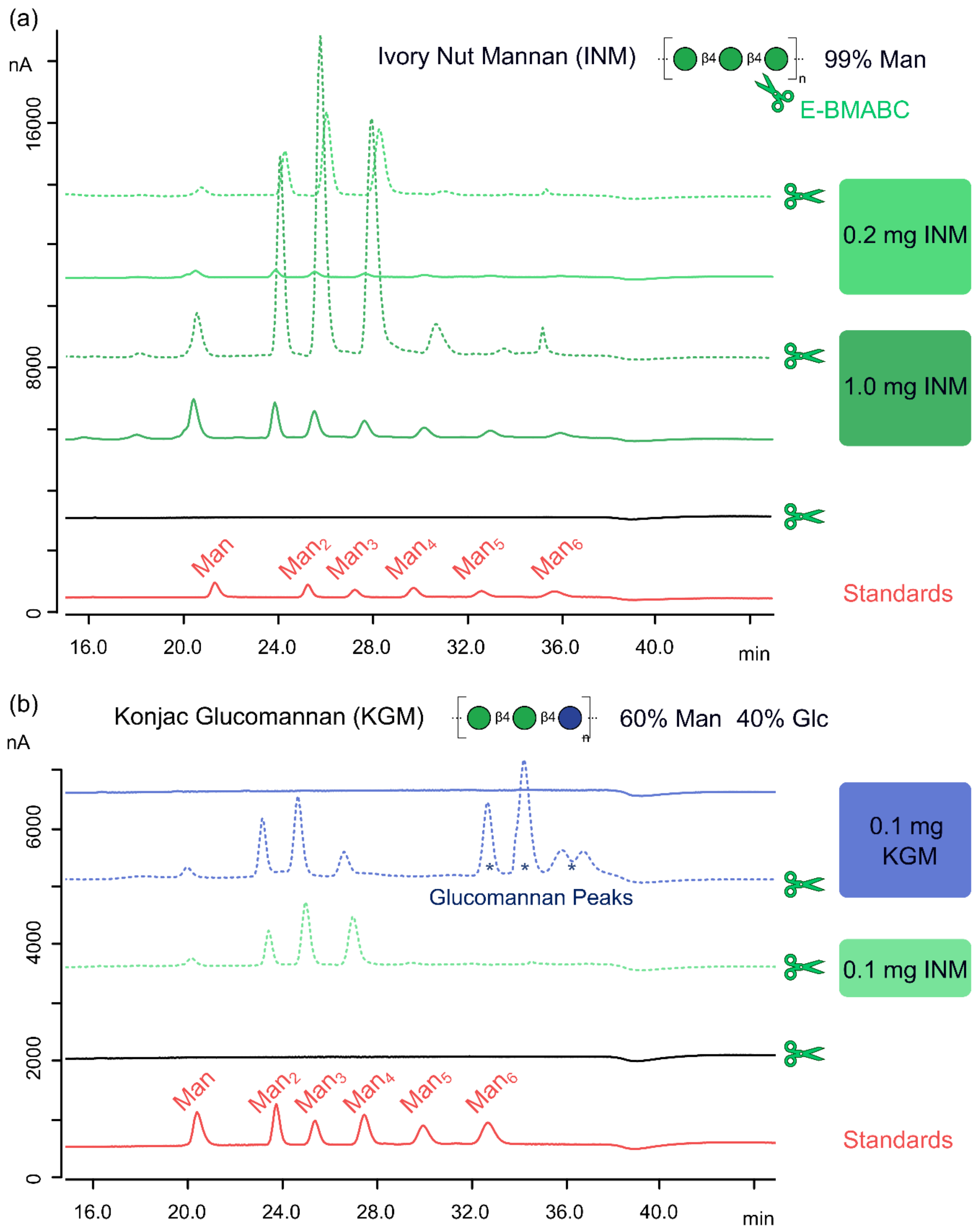

Figure S4. Oligosaccharide profiling of commercial (gluco)mannan polysaccharides. (a) HPAEC-PAD chromatograms of pure mannan polysaccharide standards with (scissors label) and without mannanase (0.1 units of E-BMABC; Megazyme) treatment. The enzyme digested at least 1 mg of INM to oligosaccharides predominantly smaller than mannopentaose (Man_5). (b) Chromatograms of ivory nut mannan (INM) and konjac glucomannan (KGM). Scissors indicate enzyme-treated polymers, or the enzyme only controls. Digested KGM shows diagnostic glucomannan peaks not digested by E-BMABC, which are absent in the INM treatments. Standards in (a) and (b) are a mixture of Man and manno-oligosaccharides (2 to 6 degrees of polymerization).

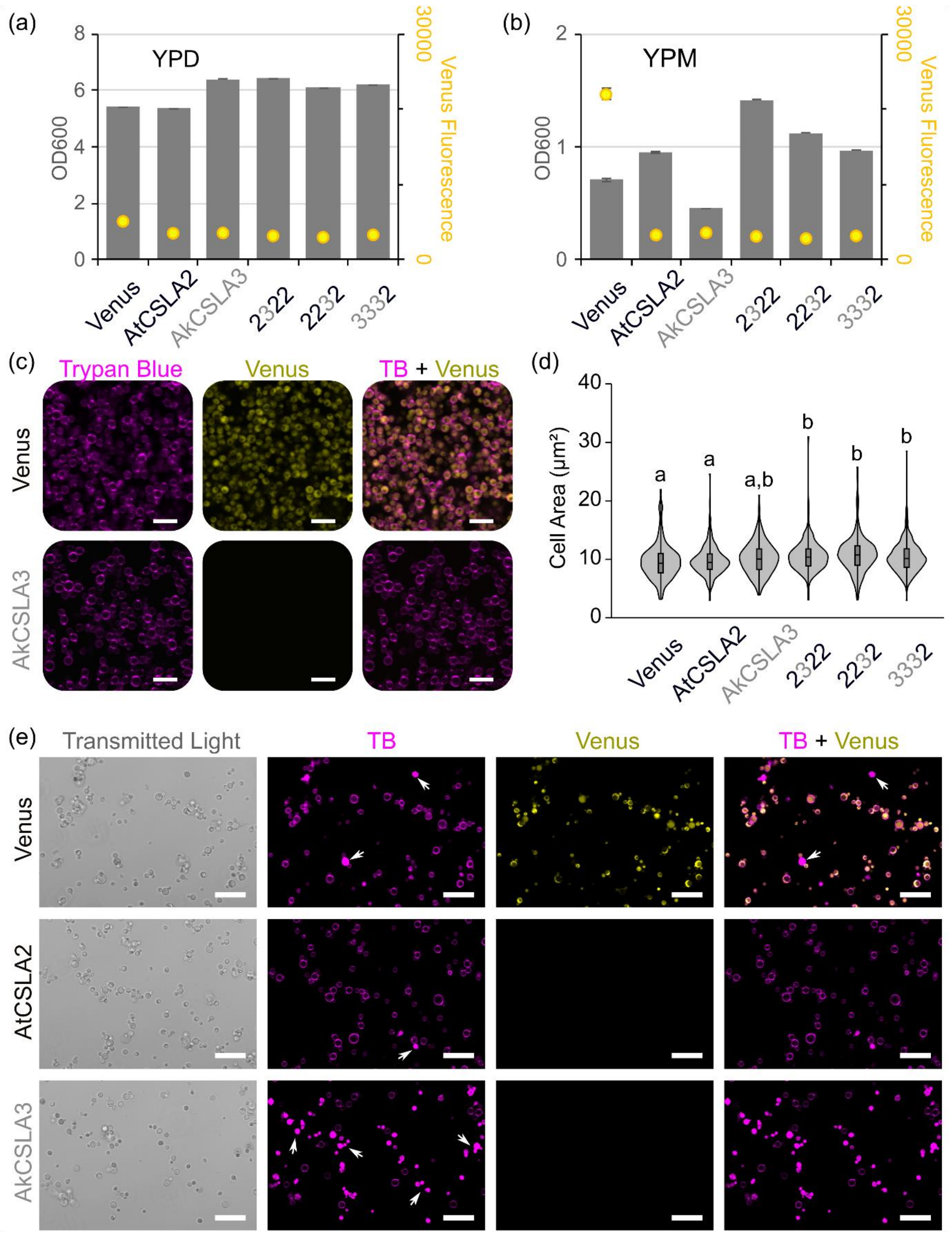

Figure S5. Yeast cell density and area after CSLA expression.
(a) YPD and (b) YPM cultivation of *Pichia* cells for 24 h. Plots show the mean + SD of two biological replicates for optical density and yellow protein fluorescence. (c) Optical sections and (d) projected area of yeast cells stained after 24 h cultivation in YPM. Violin plot shows size distribution of at least 250 cells per genotype. Different letters denote significant changes (one-way ANOVA with Tukey test, $P < 0.05$). (e) Optical sections of yeast cells stained after 72 h of cultivation in YPM + G, with glycerol to boost biomass accumulation. Arrows mark cells showing TB uptake. Scale bars = 10 µm in (c) and 20 µm in (e).

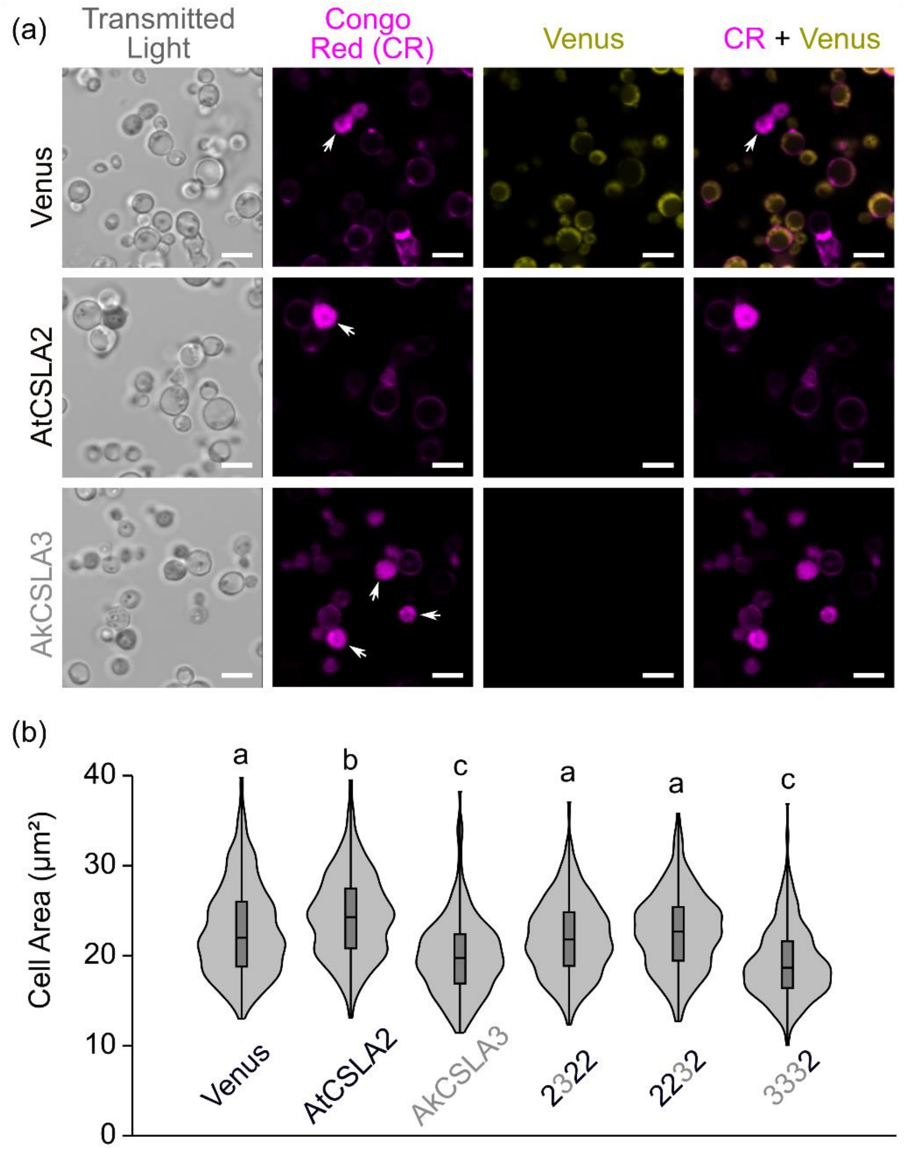

Figure S6. Imaging and quantification of yeast cells stained with Congo Red.
(a) Optical sections and (b) projected area of yeast cells stained after 72 h cultivation in YPM + G. Arrows mark cells showing Congo Red (CR) uptake. Violin plot shows size distribution of at least 350 CR-stained cells per genotype. Different letters denote significant changes (one-way ANOVA with Tukey test, $P < 0.0001$). Scale bars = 5 µm.

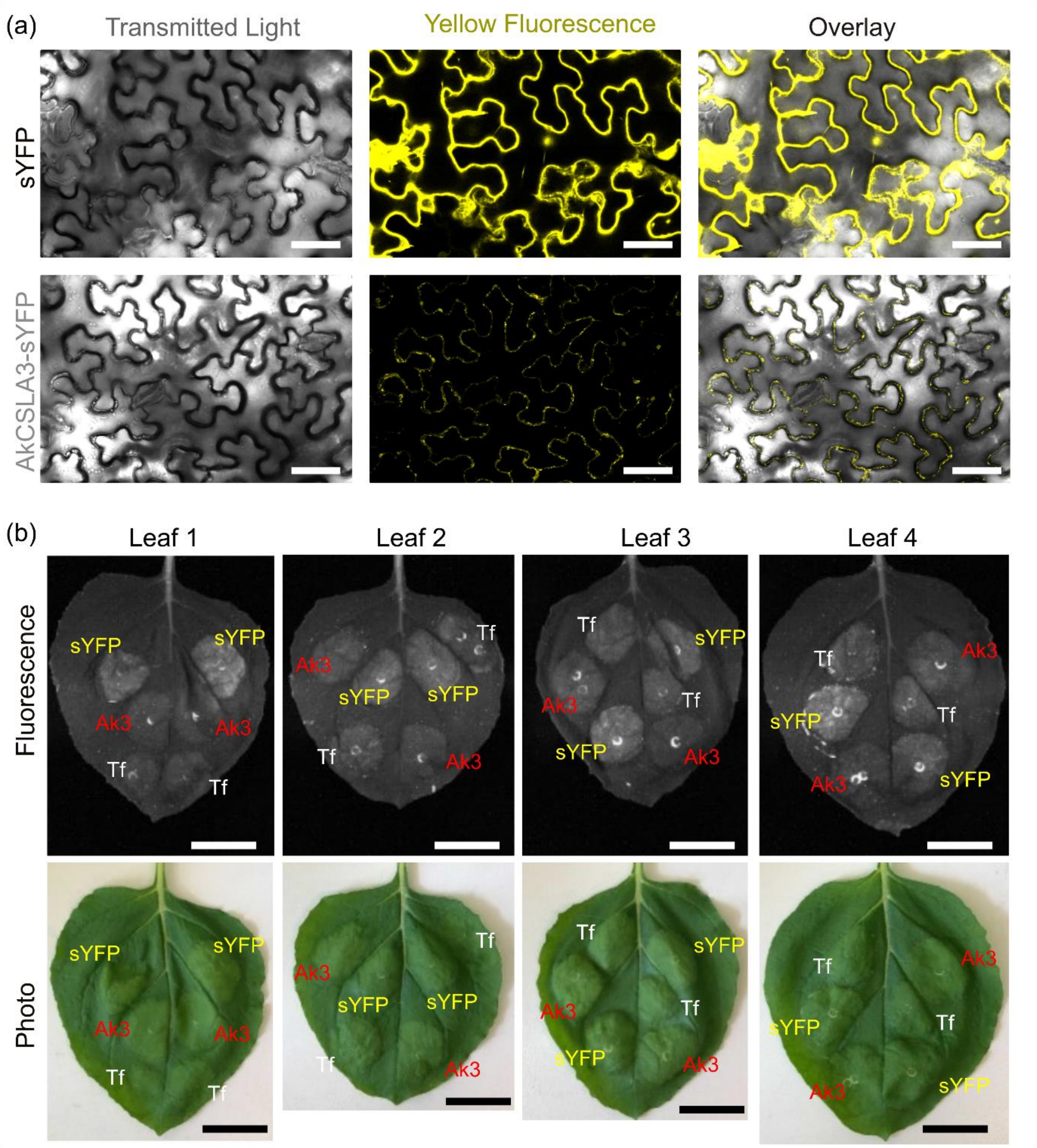

Figure S7. Expression of AkCSLA3 in *Nicotiana beanthamiana* leaves.
(a) Optical sections of tobacco cells expressing sYFP control (empty pCV01 vector) and AkCSLA3-sYFP punctae. The yellow fluorescence signals for AkCSLA3-sYFP were increased post-acquisition relative to sYFP panels. (b) Relative fluorescence protein expression of leaf spots at 6 days post-infiltration with sYFP, AkCSLA3-sYFP (labelled Ak3 in the images) or the fenugreek galactomannan galactosyltransferase TfGMGT (abbreviated Tf). None of the leaf spots showed signs of cell death. Scale bars = 50 µm (a) or 4 cm (b).

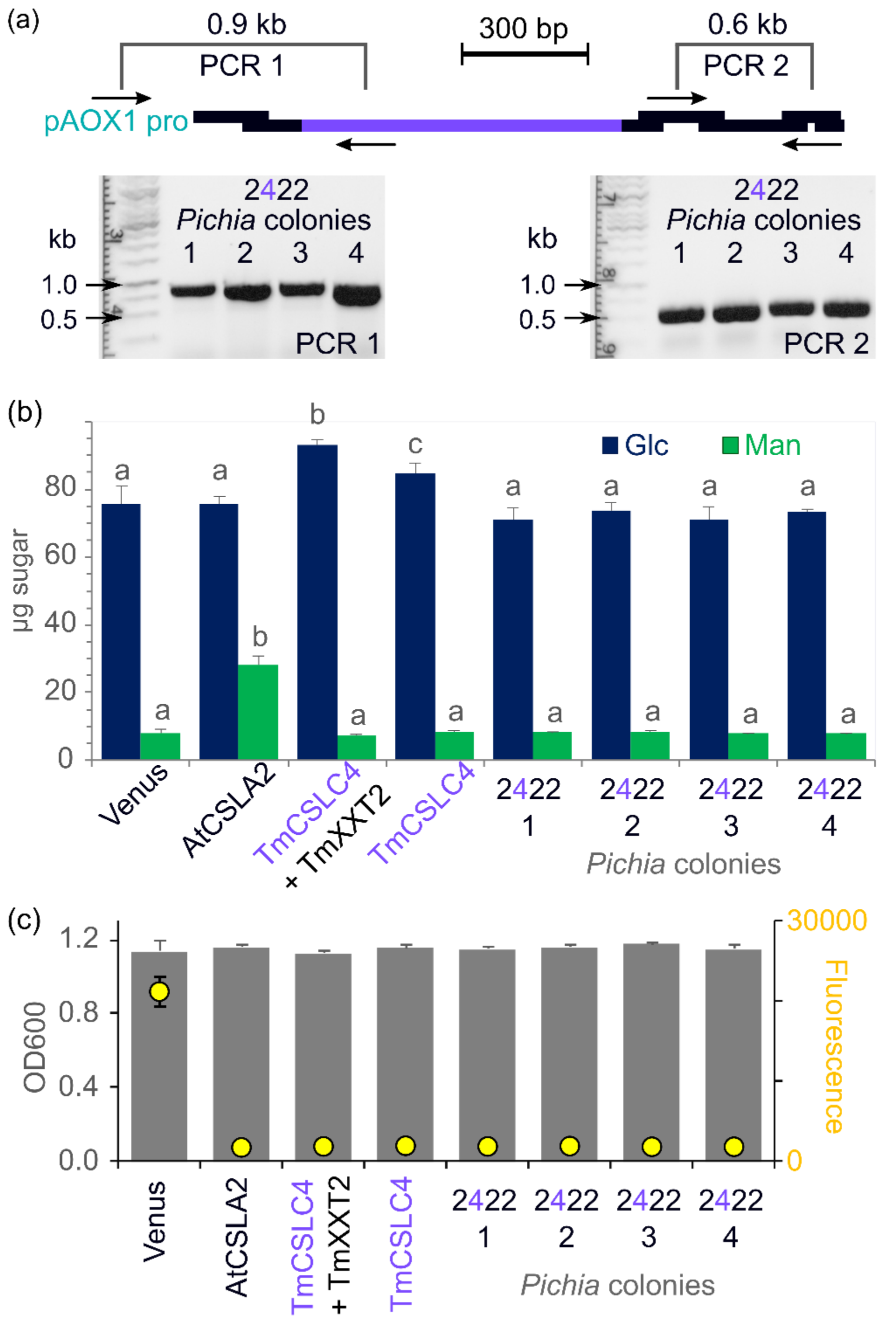

Figure S8. Replacement of a CSLA catalytic domain with that of a CSLC.
(a) The GT-containing region of AtCSLA2 was replaced with that of TmCSLC4. PCR with two primer pairs were used to unambiguously confirm the stable integration of the 2422 construct in multiple *Pichia* colonies. (b) Absolute monosaccharide composition of AKI polymers of the parental controls, and four independent 2422 strains. The plot shows the mean + SD of three biological replicates, each measured in duplicate. Different letters denote significant changes (one-way ANOVA with Tukey test, $P < 0.05$). (c) Raw optical density (OD600) and yellow protein fluorescence of 1:12 dilutions of the *Pichia* cultures from (b). Data show mean + SD of three biological replicates for each construct.

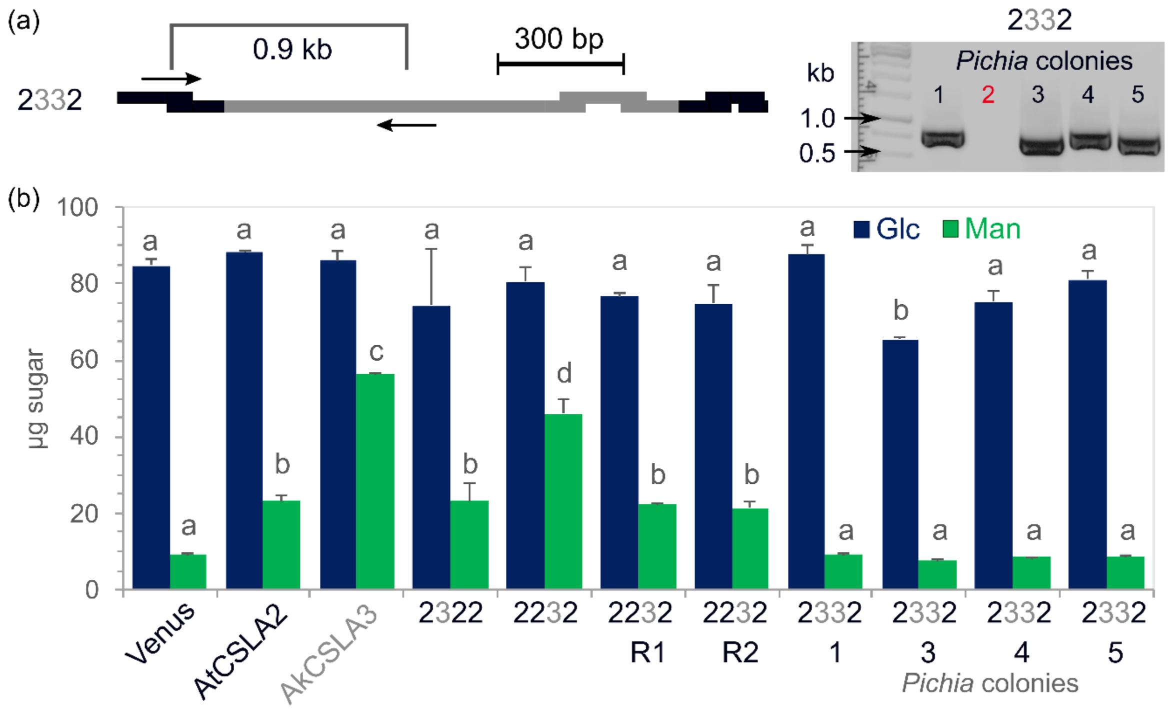

Figure S9. Combinatorial effect of AkCSLA3/AtCSLA2 domain swaps.
(a) Swap of second and third domain of AkCSLA3 into AtCSLA2 parent (2332). The 2332 construct was integrated in four of the five tested *Pichia* colonies. (b) Monosaccharide composition of AKI polymers made by positively genotyped colonies for the 2332 swap and controls. For the 2232 swap, two new colonies (R1 and R2) were obtained by re-transforming the linearized plasmid in wild-type *Pichia*. The plot shows the mean + SD of two biological replicates. Different letters denote significant changes (one-way ANOVA with Tukey test, $P < 0.05$).

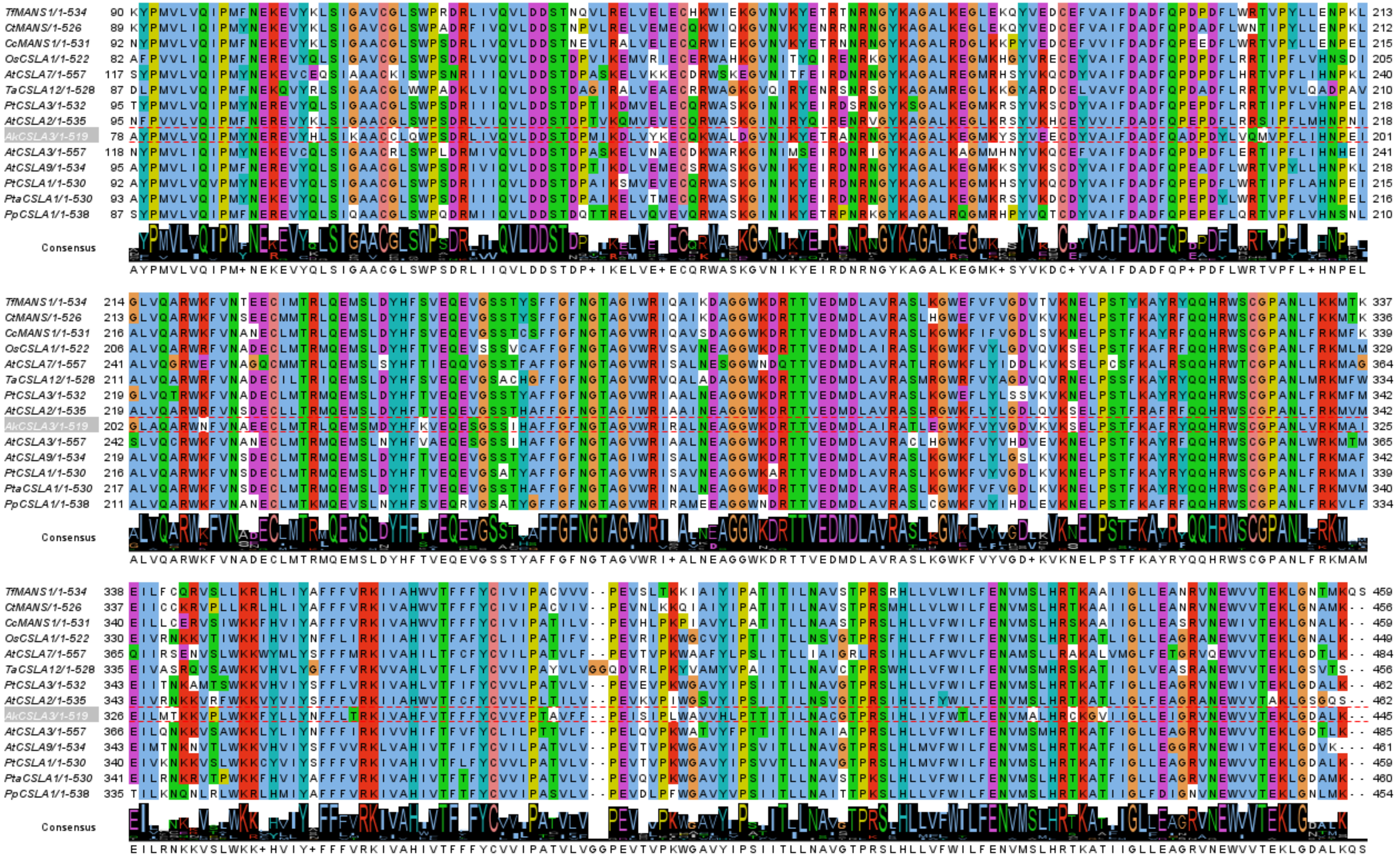

Figure S10. Alignment of active mannan and/or glucomannan synthases. The AkCSLA3 sequence is highlighted. Recombinant proteisn for the sequences above it produced relatively pure mannan with low Glc incorporation, relative to the glucomannan synthases below. Alignment was created using the MUSCLE algorithm in MEGA X. Jalview was used to visualize conserved amino acids (colored based on properties using Clusterx) and concensus sequences. N and C-terminal regions are not shown.

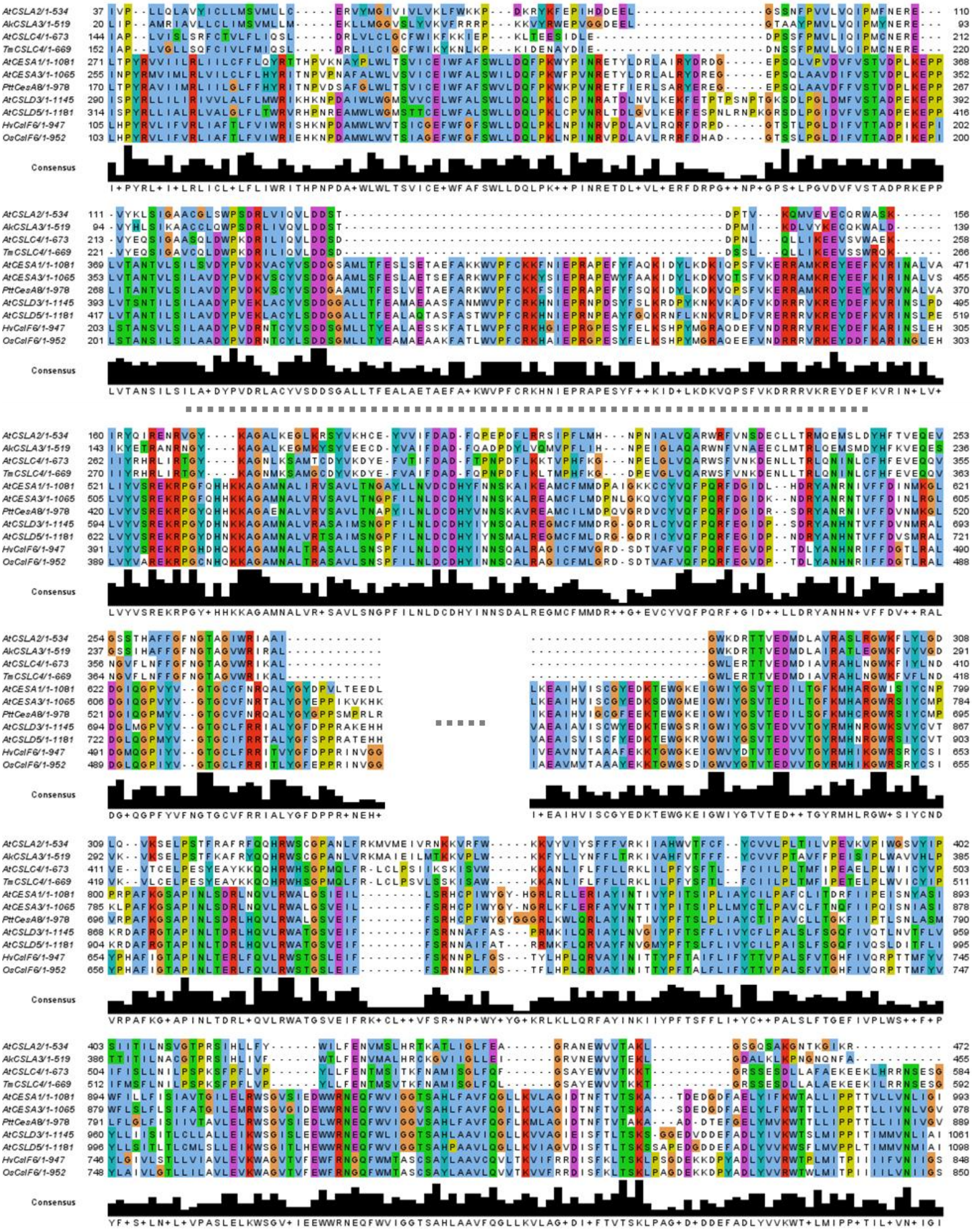

Figure S11. Alignment of CesAs and CSL representatives.
Alignment was created using the MUSCLE algorithm in MEGA X. Jalview was used to visualize conserved amino acids (colored based on properties using Clusterx) and concensus sequences. N-terminal, C- terminal, and highly variable internal regions (found in CesAs; missing in CSLAs) are not shown (marked by dashed lines). New abbreviations: *Hv*, *Hordeum vulgare*; *Os*, *Oryza sativa.*

	Primers	5' to 3' sequence (fusion sites)
Golden Gate Assembly	AtCSLA2 dom1R	atat**GGTCTC**tGAgaTGCTCGGACGGCGAGAT
	AtCSLA2 dom2F	ataa**GGTCTC**atcTCTTCGCGGCTGGAAAT
	AkCSLA3 dom1R	cgac**GGTCTC**tAtACCTTCACGTACAGGCTCA
	AkCSLA3 dom2F	ataa**GGTCTC**aGTaTTCCGCCGGCGGCCCAA
	AkCSLA3 dom2R	cggc**GGTCTC**tTtTCATATTTTATGTTCACCCCATCC
	AkCSLA3 dom3F	acat**GGTCTC**aGAaACCAGGGCCAACAGAAATG
	AkCSLA3 dom3R	atca**GGTCTC**tGtCCGATCTCCGGGTTGTGGAT
	AkCSLA3 dom4F	atat**GGTCTC**aGGaCTCGCTCAGGCTCGCTGGAA
	AtCSLA2-1F	acat**GGTCTC**aCATGGACGGTGTATCACCAAAGT
	AtCSLA2-1R	cttc**GGTCTC**tGAGGACGACGGGGAAATT
	AtCSLA2-2F	taca**GGTCTC**aCCTCCCTCGTACAAATCCCCAT
	AtCSLA2-2R	cgtc**GGTCTC**tACCATCTATGTTGCTGA
	AtCSLA2-3F	acgc**GGTCTC**tTGGTCTTGTGGACCT
	AtCSLA2-3R	atca**GGTCTC**tCGTTCGTTAGCCCTTCCTGCC
	AtCSLA2-4F	agtc**GGTCTC**aAACGAGTGGGTAGTGACT
	AtCSLA2-4R	acgc**GGTCTC**tAAGCCTAACTCGGGACATAAGTCC
	AkCSLA3-1F	ataa**GGTCTC**aCATGGCCATCGACTGGGC
	AkCSLA3-1R	cttc**GGTCTC**tGAGGACCATTGGGTAGGC
	AkCSLA3-2F	taat**GGTCTC**aCCTCGTCCAAATACCCAT
	AkCSLA3-2R	catc**GGTCTC**tACCATCGATGCTGTT
	AkCSLA3-3F	taca**GGTCTC**tTGGTCATGCGGGCC
	AkCSLA3-3R	atac**GGTCTC**tCGTTGACCCTCCCGATCT
	AkCSLA3-4F	atat**GGTCTC**aAACGAGTGGGTCGTCACA
	AkCSLA3-4R	cgcg**GGTCTC**tAAGCCTACTTTTCACTAGGAACAAAGGT
	TmCSLC4-2F	taat**GGTCTC**gCCTCgtccaaattcctatgtgc
	TmCSLC4-2R	catc**GGTCTC**gACCAccatctatgttgttgctt
pCV01	AkCSLA3 LIC F	tagttggaataggttcATGGCCATCGACTGGGCGA
	AkCSLA3 LIC R	agtatggagttgggttcacCTTTTCACTAGGAACAAAGGTG
	TfGMGT LIC F	tagttggaataggttcATGGCTACTAAGTTCGGTTCCA
	TfGMGT LIC R	agtatggagttgggttcacTGGAGAAGCAGCTGGGTAACC
Genotyping and Sequencing	At2-1F	GAGGATGGAGATCACAGGCCAA
	At2-2R	TGAAGGTCACCGAGGTAGAGAA
	At2-3F	TGGACCTGCAAATCTCTTTAGGA
	At2-4R	CCAGCCCACTGATGAAGAAA
	Ak3-1F	CCTCTACATTTGGGGTCAGG
	Ak3-2R	TCCCACGTAGACAAACTTCCAACC
	Ak3-3F	CGTAGCTCATTTCGTCACCT
	Ak3-4R	AGGTGCCAACATAACCAAGTCC
	TmCSLC4R	GCCTTGATTCTCCAAACACC
	TmCSLC4F	ACTTTCTGCTCAGGGTGTCC
	TmXXT2R	CATTGTGACCTCTGCTCGTC
	M13 R	CAGGAAACAGCTATGAC
	M13 F	TGTAAAACGACGGCCAGT

Table S3. Primer sequences used in this study.
Primers are clustered based on their primary purpose. Primer names indicate the target sequence (according to the four-domain scheme in Fig. 1). Enzyme recognition sites are in bold. Start and stop codons are underlined, and fusion sites are colored. Abbreviations: dom, domestication; F and R, forward and reverse orientation. For dom primers, lowercase bases in fusion sites are used to domesticate codons that contain unwanted cut sites.
